# On Diseases of the Kidney

**Published:** 1853

**Authors:** George Johnson


*On Diseases of the Kidney.*—By Dr. George Johnson.

[Dr. Johnson correctly remarks, how very frequently acute renal diseases, with dropsy and albuminous urine, are preceded by chills upon the surface, produced often by working or standing in wet clothes. The same in cases occurring after scarlatina: hence it is, that the chief means of preventing consequences ensuing after scarlet fever, are protection from cold until the period of desquamation has passed by,]

A consideration of the circumstances under which these attacks of acute renal disease occur, and of the appearances observable in the urine and in the kidneys, warrants the inference, that the renal changes are the result of an effort to eliminate some abnormal products, which have been conveyed to the kidneys by the blood. In cases of scarlatina, for instance, it is probable that exposure to cold has the effect of checking the process by which the fever-poison, or ferment, or the product of fermentation, is eliminated from the skin, and that the morbid materials are then transferred to the kidney, in accordance with the well-known tendency to vicarious action of the skin and kidneys. An instructive illustration of this principle is afforded by the fact of several medicines, such as, for instance, the acetate or citrate of ammonia, acting either as diaphoretics or diuretics, according as the tendency is imparted to them by the skin being kept warm, or the contrary. These facts may assist our comprehension of what probably happens when a patient is exposed to cold too early after the onset of scarlatina. The work of elimination is then transferred from the skin to the kidney; and, as a result of this, there is, in some instances, a free desquamation of the renal epithelium, analogous, as it appears, to the cutaneous desquamation which follows the eruption of scarlatina. That the cells of renal epithelium which are thus freely shed do actually carry with them a portion of the abnormal material with which the blood is infected, is, I think, as nearly certain as any position which is chiefly based upon probable evidence can be. In the urine of a girl named Robinson, who died in King's College Hospital in the year 1847, there were numerous cells of renal epithelium completely filled with crystals of oxalate of lime, while other cells contained oil; the urine was highly albuminous, and had in it many tube-casts. In this case, it is evident that the renal cells were conveying away abnormal products. The hypothesis which assumes, that the free shedding of epithelium from the uriniferous tubes is a means of eliminating morbid materials, is quite consistent with all the facts of the case, which cannot, I think, be said of any other explanation of the phenomena which has been suggested.

The process of renal desquamation, although primarily and essentially beneficial in its tendency, is not unproductive of mischief; for the secreting structures of the kidney being arranged in the form of minute tubes, which are of great length, and very tortuous, it is evident that the epithelial cells which are thrown off cannot escape with that rapidity with which the epidermic scales are cast off from the cutaneous surface; the consequence is, that the cells, and the fibrinous materials which accompany them, frequently fill, distend, and obstruct the tubes, and thus greatly impede, if they do not entirely arrest, the secretory process. And thus it happens, that many tubes being rendered inefficient, either for the further elimination of the morbid products, or for the discharge of their normal secretory functions, the urine is greatly diminished, in quantity, and the patient may die from some of the secondary consequences of an accumulation of poisonous excrement in the blood.

To illustrate these phenomena by an analogy, I would remark, that the relief from urgent symptoms which is afforded by the appearance of the small-pox eruption, is evidence, as we suppose, that some mysterious poison has been thrown from the blood into the skin. The non-appearance of the eruption is often associated with the most rapidly-fatal symptoms; and, on the other hand, the patient may be killed by the very abundance of that eruptive process which nature intended, and which was, indeed, essential for his preservation.

In like manner, it is a matter of almost daily observation, that those cases of acute dropsy with albuminous urine are, in general, most certainly and easily cured, in which there is a copious renal desquamation; while the most rapidly fatal case which I have met with, was one in which there was neither desquamation nor fibrinous effusion into the kidney-tubes.

*Med. Times and Gazette*, July 16, 1853, p. 53.

